# Time-Series Analysis of Continuously Monitored Blood Glucose: The Impacts of Geographic and Daily Lifestyle Factors

**DOI:** 10.1155/2015/804341

**Published:** 2015-03-29

**Authors:** Sean T. Doherty, Stephen P. Greaves

**Affiliations:** ^1^Department of Geography & Environmental Studies, Wilfrid Laurier University, 75 University Avenue West, Waterloo, ON, Canada N2L 3C5; ^2^Institute of Transport and Logistics Studies, The University of Sydney Business School, University of Sydney, Sydney, NSW 2006, Australia

## Abstract

Type 2 diabetes is known to be associated with environmental, behavioral, and lifestyle factors. However, the actual impacts of these factors on blood glucose (BG) variation throughout the day have remained relatively unexplored. Continuous blood glucose monitors combined with human activity tracking technologies afford new opportunities for exploration in a naturalistic setting. Data from a study of 40 patients with diabetes is utilized in this paper, including continuously monitored BG, food/medicine intake, and patient activity/location tracked using global positioning systems over a 4-day period. Standard linear regression and more disaggregated time-series analysis using autoregressive integrated moving average (ARIMA) are used to explore patient BG variation throughout the day and over space. The ARIMA models revealed a wide variety of BG correlating factors related to specific activity types, locations (especially those far from home), and travel modes, although the impacts were highly personal. Traditional variables related to food intake and medications were less often significant. Overall, the time-series analysis revealed considerable patient-by-patient variation in the effects of geographic and daily lifestyle factors. We would suggest that maps of BG spatial variation or an interactive messaging system could provide new tools to engage patients and highlight potential risk factors.

## 1. Introduction

Type 2 diabetes is known to be associated with a variety of environmental, behavioral, and lifestyle factors that have an influence on blood glucose (BG) levels. The two most widely studied factors are food consumption and physical activity [1–3]. Closely observing BG response to these factors* over time* has been shown to be an effective way of exploring possible BG management interventions.

New opportunities for exploring BG fluctuation are afforded by continuous blood glucose monitoring systems (CGMS) capable of estimating and logging second-by-second BG values and improving glycemic control in patients with diabetes [4–6]. At the same time, similarly capable technologies for passively tracking human activity and mobility patterns are emerging and capitalizing on wearable sensors such as accelerometers, heart-rate monitors, and global positioning system (GPS) tracking [7]. Combining CGMS with such technologies could provide new opportunities for exploring correlates of BG fluctuation over time and space under real-world conditions, beyond just food and exercise. This could include the effects of a wider variety of activities (e.g., shopping, leisure), travel outside the home, and exposure to different locations/environments, most of which have scarcely been explored to date.

This paper utilizes data from a unique study of 40 patients with diabetes that provided such data, including continuous monitoring of BG, food/medicine intake, location, and patient activity (exercise, travel, etc.) over a 4-day period [8]. Building on previous spatial analysis [9], this paper utilizes traditional analysis using linear regression and more disaggregate analysis using time-series models on a patient-by-patient level to explore how BG varies throughout the day. The time-series models are further distinct from previous efforts given the fine temporal (5-minute epochs) and spatial scale utilized. The implications for diabetes management and future patient management/decision support systems are discussed in the conclusions.

## 2. Materials and Methods

### 2.1. Patient Monitoring System

The patient monitoring system comprised a set of wearable sensors combined with a smartphone to transmit data to a central server for processing. The sensors include a GPS receiver, 3-axis accelerometer, and a continuous glucose monitor system (Medtronic CGMS© System Gold, Minneapolis, MN). Additionally, patients completed a food/medicine diary and a prompted-recall travel/activity diary for the duration of the study. Each of these methodological components is described in full detail in Doherty and Oh [8].

The CGMS provides automatic measurement of interstitial fluid glucose (millimoles/liter, mmol/L) every 5 minutes for 72 hours using a sensor inserted under the skin. Real-time measurements were not viewable by patients. Calibration by finger stick blood glucose is required four times per day, done manually, and entered into the device by the patient. Data were manually downloaded from the device at the end of the study period.

The GPS provided a patient's location (longitude and latitude) and speed on a second-by-second basis, typically to within 5–10 meters accuracy. The prompted recall activity diary was an interactive web-based interface that first utilized the GPS data to automatically determine the basic skeleton of a person's day (start/end time of stationary activities and movements, travel modes, and locations) and then engaged patients to fill in gaps and/or provide more details on activity types (working, shopping, physical activity, etc.), location names, and involved persons at the end of the day [10]. The 3-axis accelerometer was used to provide further detail on the intensity of physical activity by measuring acceleration in three directions 15 times per second. A combined “vector magnitude” of motion was calculated as the square root of the sums of squared directional values. Lastly, the food/medicine diary was a pocket-sized booklet completed by hand, including the types, amounts, and timing of all food, beverages, and medicines consumed.

### 2.2. Sample

A study utilizing the above system was carried out with 40 type 2 patients with diabetes from the Toronto Rehabilitation Institute. Participants were recruited via advertisements and word-of-mouth and were given incentives valued at $30 CAD. The study was approved by the Institute's Research Ethics Board. A 72-hour monitoring period was chosen, covering a four-day period. On the first day, participants came in for equipment setup, resulting in a partial day of monitoring. The next two days were full days of monitoring, followed by a fourth day when they returned to the clinic for debriefing, again resulting in a partial day of monitoring.

Of the 40 original participants, 34 ended up providing complete 72 hours of data, including requisite manual calibration of the CGMS four times daily. Two participants ceased CGMS monitoring after day 2 reporting irritation with the tape-on sensor, and four other patients' GPS data sets were deemed unusable owing to an early technical problem. The 34 participants ranged in age from 32 to 75 (mean of 56), weighed between 45 and 147 kilograms (mean 88.4), and included equal numbers of males and females. The duration since diagnosis with type 2 diabetes ranged from 3 months to 46 years (mean 9.5 years). As part of their rehab program, participants were already engaged in exercise, diet, and education programs, and most of them were actively engaged in managing their daily BG levels through a controlled program of medication including a variety of slow, medium, and fast acting insulin medications. Note that levels of reported exercise over the study period were very low (averaging one hour for most, none at all for six participants), which should be borne in mind when interpreting findings.

### 2.3. Analytical Methods

Two distinct types of analysis were performed. The conventional approach is to aggregate variables such that there is one value per participant, adhering to the statistical assumption of independent observations. Under this approach, mean BG per participant over several aggregated time periods (all 3 days, daily, or every 12 hours) was modeled using stepwise linear regression as a function of the range of explanatory variables listed in Table 1. Acknowledging the modest violation of independence, we report the 12-hour aggregation results (170 observations from 34 subjects). Note also that the natural logarithm of BG was used to mitigate heteroskedastic (unequal variance) impacts.

The collection of 5-minute data from the CGMS provided the opportunity for a much finer grain of analysis, yielding up to 864 observations per participant (28,994 in total). Over these much shorter epochs, successive BG measurements are certainly highly (auto-) correlated thus more severely violating the assumption of independent observations required of statistical techniques such as regression. Alternatively, time-series approaches such as autoregressive integrated moving average (ARIMA) have become a popular choice for dealing with such data, because of how they deal with autocorrelation issues. They are used when data are measured repeatedly at equal intervals of time and are normally applied on single participant data. They have long been used in the study of chronic diseases [11] and alongside GPS tracking to explore people's exposure to air borne pollutants [12]. Past diabetes applications have typically used daily BG values as the basis of the series [13, 14], capturing response to cyclic/harmonic patterns, interventions, gradual effects, delayed effects, and other factors.

ARIMA models predict a dependent variable's present value based on its past values plus values of other explanatory variables. This technique weighs more heavily on observations that are nearer to the point in which an independent variable is introduced, rather than based on aggregated values across a whole day or longer. Crabtree et al. [11] emphasize that “*the results of analysis by time series can reveal associations between variables in naturalistic settings where there are many confounding variables, including the variety of interactions between the individual and his/her environment which make determining causality extremely difficult*” (p. 242). Ridenour et al. [14] further suggest that ARIMA modeling holds “*promise for rigorous prevention research that uses within-person, small sample, or case study methods*” (p. 267), such as those here.

While readers are referred to texts [15, 16] and relevant journal articles [12, 17] for technical details, we take an applied approach to present the ARIMA model results, rather than focus on the mathematical structure or predictive power of the models. For the purposes of the analysis, time-series expert modeler in SPSS version 20 was used. Multivariate models (employing a transfer function) were developed for each participant to test the effects of the various variables or “interventions” as listed in Table 1. Separate models for waking hours only (6:00 a.m. to midnight) versus all hours of the day were also explored. Similarities and differences across participants were then explored.

## 3. Results

### 3.1. Aggregate Modeling: Correlations and Linear Regression

As a preliminary step, Table 2 presents bivariate correlations between mean BG and all explanatory variables. While the correlations are relatively weak, there are indications that BG levels tend to be higher during the day and increase with measures indicative of being away from home (distance from home, time in a car, and out of home time) versus being at home. Perhaps surprisingly, there were no significant correlations with food and medicine. Insights are also impacted by outliers; for instance, the suggestion that time spent with other people is significant is largely driven by one participant as was the case to a lesser extent with distance from home (see Table 2 note).

Linear regression allows a better assessment of the combined impacts of all explanatory variables on BG. Stepwise linear regression was used to arrive at a best-fit model as assessed through overall model *R* ^2^ values. In the original model, only distance from home emerged as significant, but as noted above, this is largely down to one outlier. Removing this person gives a model with time of day, time spent with people, and exercise time as significant (Table 3(a)) with the negative coefficients indicating that increased exercise time and night time periods tend to be associated with lower mean BG, while more time spent with people increases BG. On close examination, the significance of the time spent with other people was discovered to be the result of one single (outlying) participant. Once removed, this variable was no longer significant in the overall model, as shown in Table 3(b).

Caution should be exercised in interpreting the results as indicating food and medicines are nonsignificant in affecting BG levels. It must be stressed that these are patients primarily managing BG levels through a controlled program of medication, so we would expect over an average period of 12 hours for the effects to be largely cancelled out. Similarly, the fact that other variables do not emerge as significant is not indicative of them being not important, because there was relatively little variability between participants in most of the measures considered. A more general point is that it appears risky to try to analyze patients together in this way, because there is clearly great variability in explanatory factors that is not being captured through an aggregate analysis as shown by the poor *R* ^2^ values. With bearing this in mind, we turn our focus to a patient-by-patient level of analysis.

### 3.2. Disaggregate Modeling: ARIMA

A total of 68 ARIMA models were developed, given the 34 participants and two main types of models (all day versus only waking hours). An example of ARIMA model for one participant is shown in Table 4. By way of interpretation, current BG levels are primarily influenced by BG levels for the previous four epochs/lags, which in this case are 20 minutes, and to a lesser extent by exercise time and time in an automobile. Food consumption and medicine-related variables had no significant effect on BG for this participant. Across all participant models, the number of significant explanatory variables ranged from 0 to 6, averaging 2.4 (which included previous BG readings). This average increased to 4 if variables entering at more than one lag value were added. The mean *R* ^2^ values were 0.52 for all day models and 0.58 for waking hours only models (a slightly improved fit), ranging from 0.18 to 0.80 and from 0.17 to 0.87, respectively. Taken together, this suggests that participants differ markedly in the pattern of BG and extent to which current BG levels are influenced by previous readings and explanatory variables.

A summary table of the frequency with which each explanatory variable ended up being significant in the ARIMA models of all participants is shown in Table 5. Note that these factors are only marginally significant, as the majority of variability in BG readings is explained by the lag effects of previous BG readings (as intuitively expected). Additionally, the frequency of variable significance is not necessarily indicative of a variable's importance per se, because some (particularly exercise) were observed in low quantities for this sample as highlighted earlier. Nevertheless, it is interesting that indicators of travel, being away from home and conversely being at home, are all associated with significant changes in BG levels, whereas traditional variables related to food and medications were not as frequently significant. This could reflect the earlier point that participants are successfully managing the impacts of food via medications (leaving little change in BG to be detected as significant in the model), but not the other factors that remain.

## 4. Discussion and Conclusions

The analysis in this paper demonstrates that, overall, the use of smartphone-based sensors, coupled with CGMS, has the ability to reveal new geographic/lifestyle-related correlates of BG at a very fine scale, but that the impacts of these on BG fluctuation are highly personal. The more traditional aggregate analysis of mean BG over 12-hour periods revealed correlations with specific activities and travel modes, distance from home, and time of day, whilst correlations with food, medicine, and exercise were not as significant. In further exploration, if certain participants were removed, the significance of the results changed dramatically, obviously a function of the small sample size, but more pertinently providing further justification as to why disaggregate individual level analysis is warranted. Applying correlations and linear regression in an aggregated fashion can also mask associations, as data is averaged over a long period of time and thus does not adequately account for the time-lagged impacts of factors such as medications, food, and exercise that can persist for long periods of time after their occurrence (and in the case of insulin medications will vary substantially given the variety of slow to fast acting types taken by subjects in this sample). Aggregation also yields small datasets that are especially sensitive to outlying values, as shown in the analysis.

The more disaggregate time-series analysis using ARIMA modeling on a patient-by-patient basis utilized the data more fully and by design was more amendable to capturing common time-lagged affects. The ARIMA models predicted each participant's 5-minute BG value based on past values plus values of other explanatory variables, weighing more heavily on nearer observations rather than aggregated values across a whole day. The expectation that ARIMA modeling would reveal a wider variety of associations in naturalistic settings [11] even with a small sample [14], was met, as a much more specific and interesting mix of correlating factors was discovered on a patient-by-patient basis. On average, this included 2-3 explanatory variables per participant, most often related to the conduct of specific activity types or certain locations (especially at home or shopping), travel by different modes such as automobile and transit, or being out of home (especially far from home). Traditional variables related to food intake and medications were again less often significant in explaining BG fluctuation. This suggests that participants were successfully managing the impacts that food has on BG using their medication regime but were not actively controlling for the impacts of these other geographic/lifestyle-related factors.

Taken together, whilst the aggregated correlation and regression analysis revealed many of the same correlating factors as the ARIMA modeling, conducting the aggregated analysis in isolation may lead to the temptation to over-generalize to a population, whereas the time-series analysis revealed considerable patient-by-patient variation in terms of the effects of geographic and daily lifestyle factors. At the same time, we acknowledge the limitations of the study. Clearly, this was a small sample of a very specific segment of the population, and the intent was never to generalize the findings. Rather, the purpose was to demonstrate the potential value of both personalized monitoring and disaggregate analysis for the management of health care and hopefully encourage others to pursue similar lines of enquiry in the future. We also acknowledge that our decision to treat each patient as (in effect) a separate time-series model, while providing unique intra-patient insights, limited the analysis of interpatient effects at the disaggregate level. An alternative approach is to pool the time-series data, creating one series for analysis. Such an approach has been used in cohort studies similar to this one [18], although it does bring with it a separate set of challenges and should only be applied subject to various statistical constraints [19].

Given this, the practical implications of these findings are worth considering. What seems clear is that generalized suggestions on the management of geographic/lifestyle factors that impact BG are likely not feasible or, in the least, should be augmented by highly personalized advice. What this will require is an effective patient management/decision support system that goes beyond tracking the data and instead provides for personalized analysis of the data to assist caregivers in both diagnosing and monitoring patient progress. In particular, as a complement to traditional temporal graphical analysis, we would suggest that maps of BG spatial variation such as those shown in Doherty [9] could provide a new tool for caregivers to engage patients in discussion of potential risk factors such as locations or activity types associated with high/low BG and engender more concerted efforts to manually monitor BG during such periods. At a more advanced level, more sophisticated time-series analysis of data could assist in identifying risk factors personalized to the patient. This approach would correspond to telemedicine's “*missing element*” [20], and with Balakrishnan et al.'s [21], call for more* personalized* BG prediction models sensitive to lifestyle interventions deemed necessary for devising optimal patient specific advice and prescriptions.

A more pragmatic enhancement to such systems would be to incorporate live interactive messaging to reach out to patients outside of face-to-face meetings with caregivers, warning of* possible* risk factors identified just for them, such as a lengthy drive, risky location, or long time/distance away from home. Associated with this could be a suggestion to manually check their BG and/or modify behavior. This would serve as a complement to traditional BG monitoring advice associated with the management of food- and exercise-related risks. Given the wide-scale proliferation of smartphones, including the developing world, this could potentially reach a wide audience.

## Figures and Tables

**Table 1:** Explanatory variables.

Variable name	Description	Value/units in given time period^a^
Calories consumed	From food and beverages	Total calories
Meds taken	Of any type	0 = no, 1 = yes
Insulin taken	Variety of slow, medium, and fast acting insulin medications	0 = no, 1 = yes
Movement intensity	Vector magnitude, square root of the sum of the squared 3-axis acceleration readings	Average vector magnitude^b^
Exercise time	Aerobic, weights, walking, and so forth	Total minutes
Trip time	Time spent travelling by any mode	Total minutes
Automobile time	Time spent travelling by personal automobile	Total minutes
Public transit time	Time spent travelling by public transit (bus, streetcar, subway, and train)	Total minutes
At-home time	Time spent at home	Total minutes
Work/school time	Time spent at work or school	Total minutes
Shopping time	Time spent shopping (grocery, clothing, etc.)	Total minutes
Restaurants time	Time spent in a restaurant of any type	Total minutes
Out-of-home time	Time spent outside the home	Total minutes
Time spent with others	Time spent with other people involved	Total minutes
Time of day	Daytime (7:00AM to 7:00PM) or nighttime	0 = daytime, 1 = nighttime
Distance home	Straight-line distance between current location and home location	Average distance in kilometers

^a^Time periods included the past 12 hours for aggregate correlation/regression analysis and over past 5 minutes for time series ARIMA modeling.

^b^Low values are typically associated with stationary activities and higher values are indicative of more physically active periods.

**Table 2:** Correlation with mean blood glucose calculated over 12-hour periods (*n* = 170, 34 participants × 5 twelve hour periods).

Explanatory variable (in last 12-hour period)	Correlation with mean BG
Movement intensity	−0.072
Calories consumed	0.071
Medicines taken	−0.015
Insulin taken	0.050
Exercise time	−0.127
At-home time	−0.240^**^
Work/school time	0.124
Shopping time	0.003
Restaurant time	0.099
Automobile time	0.169^*^
Public transit time	0.076
Time spent with others^a^	0.200^**^
Day/night	−0.204^**^
Out-of-home time	0.226^**^
Travel time	0.181^*^
Average distance from home^b^	0.363^**^

^∗^Correlation is significant at the 0.05 level (2-tailed).

^**^Correlation is significant at the 0.01 level (2-tailed).

^a^Primarily due to one participant reporting substantially more time spent with another person. If this person is removed, correlation drops to 0.055 and 0.016.

^b^Heavily influenced by one participant with an average distance of home some three times higher than the next highest. Removing this person reduced correlations to 0.228^**^ and 0.157^*^.

**Table:** (a) 33 participants (*n* = 165, *R* ^2^ = 0.176)

Variables	Standardized coefficients	*t*	Sig.
	Beta		
(Constant)		56.5	0.000
Night	−0.35	−4.09	0.000
Time spent with people in last 12 hours	0.22	2.74	0.007
Exercise time in last 12 hours	−0.23	−2.62	0.010

**Table:** (b) Two outliers removed (*n* = 160, *R* ^2^ = 0.171)

Variables	Standardized coefficients	*t*	Sig.
	Beta		
(Constant)		61.8	0.000
Night	−0.39	−4.43	0.000
Exercise time in last 12 hours	−0.31	−3.51	0.001

**Table 4:** ARIMA blood glucose modeling results, single participant (*n* = 864, *R* ^2^ = 0.612).

Variables	Lag^a^	Estimate	*t*	Sig.
Previous BG readings	Lag 1	−0.61	−18.3	0.000
	Lag 2	−0.28	−7.28	0.000
	Lag 3	−0.22	−5.71	0.000
	Lag 4	−0.27	−7.93	0.000

Exercise time in last 5 min	Lag 0	0.00	−3.75	0.000
	Lag 1	8.47*E* − 05	2.54	0.011

Trip-automobile time in last 5 min	Lag 0	5.58*E* − 05	4.07	0.000
	Lag 4	−3.49*E* − 05	−2.49	0.013

^a^Outputs are displayed similar to other multivariate models (overall *R* ^2^, variables entering model) but with the addition of one distinct feature: the time “lag” with which BG was significantly autocorrelated with the variable. A lag of 1 indicates autocorrelation of BG with the explanatory value in the preceding 5-minute interval, whilst higher lags indicate associated autocorrelation further back in time. A single explanatory variable can have multiple lagged effects, listed in sequence in the output with separate estimates.

**Table 5:** Frequency of variable significance in 5-minute ARIMA models.

Variables	All day models		Waking hours only models	
At-home time	11	(33%)	6	(18%)
Automobile time	9	(27%)	10	(30%)
Public transit time	6	(18%)	5	(15%)
Shopping time	5	(15%)	4	(12%)
Calories consumed	4	(12%)	7	(21%)
Distance home	4	(12%)	6	(18%)
Exercise time	2	(6%)	2	(6%)
Meds taken (yes/no)	2	(6%)	1	(3%)
Time spent with others	2	(6%)	3	(9%)
Restaurants time	1	(3%)	2	(6%)
Insulin taken (yes/no)	0	(0%)	1	(3%)
Movement intensity	0	(0%)	0	(0%)
Trip time	0	(0%)	0	(0%)
Work/school time	0	(0%)	0	(0%)
Out-of-home time	0	(0%)	0	(0%)
